# Cyclin-Dependent Kinase 6 Identified as the Target Protein in the Antitumor Activity of *Tetrastigma hemsleyanum*

**DOI:** 10.3389/fonc.2022.865409

**Published:** 2022-04-11

**Authors:** Chaoguang Wei, Yuxiang Zhao, Tao Ji, Yong Sun, Xudong Cai, Xin Peng

**Affiliations:** ^1^ Ningbo Municipal Hospital of TCM, Affiliated Hospital of Zhejiang Chinese Medical University, Ningbo, China; ^2^ United New Drug Research and Development Center, Biotrans Technology Co., Ltd, Ningbo, China; ^3^ State Key Laboratory of Food Science and Technology, Nanchang University, Nanchang, China

**Keywords:** CDK6, MET, antitumor, pan-cancer analysis, *Tetrastigma hemsleyanum*

## Abstract

**Background:**

*Tetrastigma hemsleyanum* (*T. hemsleyanum*) is widely used as an adjuvant drug for tumor therapy but its antitumor therapeutic targets and molecular mechanisms have remained unclear. The prediction and analysis of natural products has previously used only network pharmacology methods to identify potential target proteins from public databases. In this study, we use comprehensive bioinformatics analysis and experimental verification to determine the antitumor mechanism of *T. hemsleyanum*.

**Methods:**

Network pharmacology analysis was used to predict the potential *in vivo* target proteins of *T. hemsleyanum*. The expression matrix and clinical data to perform an analysis of hub genes were collected from the TCGA and GTEx databases, specifically the analysis of expression, prognosis, tumor immune cell infiltration analysis, immune checkpoint genes, microsatellite instability, tumor mutational burden, tumor neoantigen, and immune microenvironment, which identify the roles and biological functions of the hub genes in pan-cancer. Finally, gene set enrichment analysis was used to verify the biological processes and signaling pathways involved in the pan-cancer expression profile.

**Results:**

We found 124 potential *in vivo* target proteins of *T. hemsleyanum* through network pharmacological analysis, and five hub genes (AKR1C1, MET, PTK2, PIK3R1, and CDK6) were then screened by protein–protein interaction (PPI) network analysis and molecular complex detection analysis (MCODE). Experimental intervention with an aqueous extract of *T. hemsleyanum* verified that these hub genes are the target proteins involved in the regulation of *T. hemsleyanum* in cells. A pan-cancer analysis then confirmed that CDK6 and MET are potential targets upon which *T. hemsleyanum* may exert antitumor action, especially in ACC, CESC, LGG, and PAAD. The CDK6 protein targeted by *T. hemsleyanum* is also involved in the immune and mutation process of pan-cancer, especially in the regulation of immune cell infiltration, immune checkpoint gene expression, microsatellite instability, tumor mutation burdens, and tumor neoantigens. Together, these analyses show that *T. hemsleyanum* affects tumor immune regulation and genomic stability. Finally, a gene set enrichment analysis confirmed that *T. hemsleyanum* regulates the cell cycle checkpoint.

**Conclusions:**

We found that *T. hemsleyanum* can behave as an antitumor agent by acting as a potential cell cycle checkpoint inhibitor in CDK6-driven tumors, such as ACC, CESC, LGG, and PAAD, and that it acts as a tyrosine kinase receptor inhibitor that inhibits the expression of the proto-oncogene MET. Combined with an analysis of immune and mutation correlations in pan-cancer, we determined that *T. hemsleyanum* may function biologically as an immune regulator and interfere with the stability of the tumor genome, which is worthy of further study.

## Introduction

*Tetrastigma hemsleyanum* Diels et Gilg (*T. hemsleyanum*) is a valuable medicinal plant mainly found in the eastern and southern regions of China and Southeast Asia (1). The plant is traditionally used as an herbal medicine for the treatment of hepatitis and rheumatic arthritis and for regulating immune function (2, 3). Previous research has found that flavonoids, lipid soluble substances, and polysaccharides extracted from *T. hemsleyanum* have strong antiproliferative effects on a variety of cancers (4), including leukemia and carcinomas of the lung (5), liver (6), uterus (7), and intestines (8). To date, more than one hundred compounds have been isolated and identified from *T. hemsleyanum*, including flavonoids and their glycosides, phenolic acids, and alkaloids (9), but the antitumor therapeutic targets and molecular mechanisms of the agents extracted from *T. hemsleyanum* have remained poorly understood.

The cell cycle in normal cells is finely regulated and cell cycle checkpoints prevent uncontrolled proliferation and repair any damaged DNA. Growth factors may activate cell cycle progression and are regulated by two groups of proteins: cyclins and cyclin-dependent kinases (CDK) (10). CDKs are a family of serine/threonine protein kinases in complex with cyclins that regulate various critical cellular processes, including cell cycle progression, transcription, neurogenesis, and apoptosis. Deregulation of CDKs is directly related to tumorigenesis, and the important roles of CDKs and cyclins in promoting cancer genesis and progression makes them attractive targets for drug inhibition. To date, at least 20 CDKs and 30 cyclins have been reported, and of these, emerging evidence suggests that CDK6 has a vital transcriptional role in tumor angiogenesis and thus represents a promising target for antitumor treatment (11).

In recent years, with the development of next-generation sequencing techniques and omics and the establishment of The Cancer Genome Atlas (TCGA) database, a new pipeline called pan-cancer analysis has developed to study the correlations between the expression of single genes or gene sets of at least 33 tumors and their phenotypes, such as prognosis, tumor immune cell infiltration, immune checkpoint gene expression, microsatellite instability, tumor mutational burden (TMB), and the immune microenvironment. The *in vivo* action targets of natural products can often be obtained through network pharmacological analysis and combined with the technical methods of network pharmacology and pan-cancer analysis, we can thus explore the different functions and related phenotypes of natural product targets in various tumors, which offer a new approach to screening the possible uses of natural products by mining their pharmacological mechanisms. In this study we examine, through a series of analyses, whether CDK6 is a potential target of *T. hemsleyanum* and whether *T. hemsleyanum* might have an antitumor function as a cell cycle checkpoint inhibitor.

## Methods

### Data Collection

The gene expression profile data and clinical data for pan-cancer analysis were obtained from the TCGA database. Due to the small number of normal samples in the database and in order to improve the feasibility of the study, we added normal samples from the Genotype-Tissue Expression (GTEx) database. The abbreviations for the tumors in the pan-cancer analysis are shown in **Table 1**.

**Table 1:** The abbreviations of tumors in pan-cancer analysis.

Tumor Name	Abbreviations
Adrenocortical carcinoma	ACC
Bladder Urothelial Carcinoma	BLCA
Breast invasive carcinoma	BRCA
Cervical squamous cell carcinoma and endocervical adenocarcinoma	CESC
Cholangiocarcinoma	CHOL
Colon adenocarcinoma	COAD
Rectum adenocarcinoma	READ
Lymphoid Neoplasm Diffuse Large B-cell Lymphoma	DLBC
Esophageal carcinoma	ESCA
Glioblastoma multiforme	GBM
Head and Neck squamous cell carcinoma	HNSC
Kidney Chromophobe	KICH
Kidney renal clear cell carcinoma	KIRC
Kidney renal papillary cell carcinoma	KIRP
Acute Myeloid Leukemia	LAML
Low grade glioma	LGG
Liver hepatocellular carcinoma	LIHC
Lung adenocarcinoma	LUAD
Lung squamous cell carcinoma	LUSC
Mesothelioma	MESO
Ovarian serous cystadenocarcinoma	OV
Pancreatic adenocarcinoma	PAAD
Pheochromocytoma and Paraganglioma	PCPG
Prostate adenocarcinoma	PRAD
Rectum adenocarcinoma	READ
Sarcoma	SARC
Skin Cutaneous Melanoma	SKCM
Stomach adenocarcinoma	STAD
Stomach and Esophageal carcinoma	STES
Testicular Germ Cell Tumors	TGCT
Thyroid carcinoma	THCA
Thymoma	THYM
Uterine Corpus Endometrial Carcinoma	UCEC
Uveal Melanoma	UVM

### Network Pharmacology Analysis

In previous published research, we searched for and analyzed the components of *T. hemsleyanum* (12). The Swiss Target Prediction database (http://www.swisstargetprediction.ch/) and UniProt database (https://www.uniprot.org/) were used to identify compound-related targets with probability values of 0.5 or greater and the probability value derived from a cross-validation analysis was used to rank the targets and to estimate the accuracy of the predictions.

### Protein–Protein Interaction (PPI) Network Analysis

The compound-related targets were imported into the STRING (13), BioGrid (14), OmniPath (15), and InWeb_IM (16) databases, and the files of interactions between the network nodes were obtained. Cytoscape was then used to visualize the PPI network and molecular complex detection (MCODE) was used to screen the hub nodes.

### Gene Function Enrichment Analysis

The Gene Ontology (GO) biological process project, the Kyoto Encyclopedia of Genes and Genomes (KEGG), and the Hallmark Gene Sets served as biological functioning or signaling pathway sources for conducting gene function enrichment analysis. The compound-related targets were imported into Metascape (http://metascape.org/) (17) for functional enrichment analysis and terms with a minimum count of 3, p-values below.01, and enrichment factors greater than 1.5, were used as the threshold for the enrichment analysis; the terms at the top of the selected enrichment indexes were visualized by bar graphs.

### Cell Culture

HEK293 cells (CRL-1573; American Type Culture Collection, VA, USA) were cultured in MEM medium (11090073; Invitrogen, MA, USA) with 10% FBS at 37°C in 5% carbon dioxide according to the manufacturer’s instructions. Before the experiment, the cells underwent and passed cell identification and mycoplasma detection for quality control.

### Quantitative PCR Assay

The gene expression levels of AKR1C1, MET, PTK2, PIK3R1, and CDK6 were determined by quantitative PCR. HEK293 cells were sub-packed into 12-well plates and cultured overnight; the method of preparing an aqueous extract of *T. hemsleyanum* is given in previous studies (2). The HEK293 cells were treated with 0, 150, or 300 ug/ml aqueous extract of *T. hemsleyanum* then lysed with RNA-isolating Total RNA Extraction Reagent (R401-01; Vazyme, Nanjing, China) to prepare high-purity RNA, according to the manufacturer’s instructions. HiScript III 1st Strand cDNA Synthesis (Kit R312-01; Vazyme, Nanjing, China) was used to prepare the cDNA library for reverse transcription. The -2^ΔΔCT^ method was used to quantify the gene expression levels and the experiment was repeated three times independently. Student’s t-test was used for comparative analysis, with a p-value below.05 considered statistically significant. Primer information is shown in **Table 2**.

**Table 2:** Primer information.

Primer Name	Sequence (5’→3’)	Tm (°C)
h-AKR1C1-F	TCCAGTGTCTGTAAAGCCAGG	58
h-AKR1C1-R	CCAGCAGTTTTCTCTGGTTGAA	58
h-MET-F	AGCAATGGGGAGTGTAAAGAGG	58
h-MET-R	CCCAGTCTTGTACTCAGCAAC	58
h-PTK2-F	GCTTACCTTGACCCCAACTTG	58
h-PTK2-R	ACGTTCCATACCAGTACCCAG	58
h-PIK3R1-F	ACCACTACCGGAATGAATCTCT	58
h-PIK3R1-R	GGGATGTGCGGGTATATTCTTC	58
h-CDK6-F	CCAGATGGCTCTAACCTCAGT	58
h-CDK6-R	AACTTCCACGAAAAAGAGGCTT	58
β-actin-F	CTCCATCCTGGCCTCGCTGT	58
β-actin-R	GCTGTCACCTTCACCTTCC	58

### Gene Expression Analysis

The gene expression matrix was obtained from the TCGA and GTEx databases. The expression distributions of the genes in tumor tissues and normal tissues were analyzed using a Wilcoxon test. A p-value below.05 was considered statistically significant. The results were visualized by a violin plot prepared using ggplot2.

### Gene Prognostic Analysis

Clinical data were obtained from the TCGA database and a univariate Cox regression analysis was performed on the screened hub genes. A forest map was used to display the p-value, HR, and 95% CI of each variable, prepared using the forestplot R package. Statistical analysis was performed using R software. A Wilcoxon test was used to compare the two groups of data, with a p-value below.05 considered to be statistically significant. The gene prognosis relationship, screened using the univariate Cox analysis, was verified by Kaplan–Meier survival analysis and the receiver operating characteristic (ROC) curve. The R packages survival and survminer were used to perform the Kaplan–Meier curve, logrank test, and univariate Cox proportional hazards regression analyses and the ROC curve was prepared with the R package timeROC.

### Correlation Analysis of Tumor Immune Cell Infiltration

The CIBERSORT and quanTIseq methods were used to cross-verify the correlation between the gene expression and tumor immune cell infiltration, which was performed with the immunedeconv package. A Wilcoxon test was used to compare the two groups of data, and a p-value below.05 was considered statistically significant. The correlations between selected gene expressions and tumor immune cell infiltration levels in pan-cancer were visualized by a circle plot.

### Correlation Analysis of Immune Checkpoint Gene Expression

Wilcoxon analysis was used to compare the correlations between the expression levels of the selected genes and the gene expression levels of immune checkpoints such as SIGLEC15, IDO1, CD274 (PD-L1), HAVCR2, PDCD1 (PD-1), CTLA4, LAG3, and PDCD1LG2 (PD-1), and pheatmap software was used to visualize the results. A p-value below.05 was considered statistically significant.

### Correlation Analysis of Microsatellite Instability (MSI)

Spearman’s correlation coefficients between the selected gene expression levels and MSI levels were calculated (18). The relationships between the selected genes and the MSI levels of four tumors (ACC, CESC, LGG, and PAAD) were visualized by a lollipop plot and the correlations between the selected gene expressions and MSI levels in pan-cancer were visualized by a radar plot. A p-value below.05 was considered statistically significant.

### Correlation Analysis of TMB

Spearman’s correlation coefficients between the selected gene expression levels and TMB levels were calculated (19). The relationships between the selected genes and the TMB levels of four tumors (ACC, CESC, LGG, and PAAD) were visualized by a lollipop plot and the correlations between the selected gene expressions and TMB levels in pan-cancer were visualized by a radar plot. A p-value below.05 was considered statistically significant.

### Correlation Analysis of Tumor Neoantigens in Pan-Cancer

The correlations between the selected gene expressions and neoantigen levels in pan-cancer were visualized by a radar plot. A p-value below.05 was considered statistically significant.

### Correlation Analysis of the Immune Microenvironment in Pan-Cancer

The immune microenvironment analysis of tumor tissue consisted of three parts: an ESTIMATEScore, an ImmuneScore, and a StromalScore. The correlations between the selected gene expressions and immune microenvironment levels in pan-cancer were visualized by a circle plot. A p-value below.05 was considered statistically significant.

### Gene Set Enrichment Analysis (GSEA) in Pan-Cancer

To observe the biological functions of the selected genes in the tumor, we divided the pan-cancer samples into high and low groups according to the gene expression levels. GSEA was used to enrich the KEGG pathways in the high expression group and the Hallmark pathways in the low expression group. The threshold was | NES | > 1, p <.05, FDR <.25.

## Results

### Identification of Potential Target Proteins of *T. hemsleyanum*

**Figure 1:**
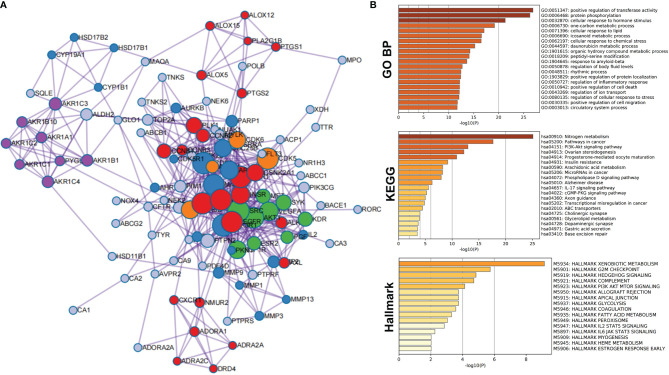
Protein–protein interaction network and enrichment analysis of network members of potential target proteins in *T. hemsleyanum*. **(A)** PPI network. **(B)** Enrichment analysis of network members of potential target proteins by GO, KEGG, and Hallmark.

As shown in **Table S1**, a total of 124 compound-related targets were found after eliminating duplicates. Enrichment analysis and PPI network analysis were performed by Metascape to explore the biological functions of the 124 targets. The complete PPI network is shown in **Figure 1A**. Compared with proteins at the edge of the network, those at the center have more connections, indicating that they may play an important biological function. All information regarding PPI members is given in **Table S2**.

PPI members were enriched and analyzed at the functional level using the GO biological process project, KEGG, and Hallmark Gene Sets, as shown in **Figure 1B**. From the GO biological processes, 455 pathways were found; ranking these enrichment pathways by log_10_ (P), the top 10 pathways were positive regulation of transferase activity, protein phosphorylation, cellular response to hormone stimulus, one-carbon metabolic process, cellular response to lipid, icosanoid metabolic process, cellular response to chemical stress, daunorubicin metabolic process, organic hydroxy compound metabolic process, and peptidyl-serine modification—the top 20 pathways are shown in **Table 3**. From KEGG, 49 pathways were enriched and the top 10 were nitrogen metabolism, pathways in cancer, PI3K-Akt signaling pathway, ovarian steroidogenesis, progesterone-mediated oocyte maturation, insulin resistance, arachidonic acid metabolism, microRNAs in cancer, phospholipase D signaling pathway, and Alzheimer’s disease; the top 20 enriched terms are presented in **Table 4**. From the Hallmark Gene Sets, 16 pathways were enriched and the top 10 were xenobiotic metabolism, G2M checkpoint, hedgehog signaling, complement, PI3K-Akt-mTOR signaling, apical junction, glycolysis, allograft rejection, coagulation, and fatty acid metabolism; details of all enriched terms are presented in **Table 5**.

**Table 3:** List of tops enriched GO terms.

GO	Category	Description	Count	%	Log_10_(P)
GO:0051347	GO Biological Processes	positive regulation of transferase activity	32	25.81	-26.85
GO:0006468	GO Biological Processes	protein phosphorylation	34	27.42	-26.25
GO:0032870	GO Biological Processes	cellular response to hormone stimulus	26	20.97	-21.32
GO:0006730	GO Biological Processes	one-carbon metabolic process	12	9.68	-19.17
GO:0071396	GO Biological Processes	cellular response to lipid	23	18.55	-17.08
GO:0006690	GO Biological Processes	icosanoid metabolic process	14	11.29	-16.56
GO:0062197	GO Biological Processes	cellular response to chemical stress	18	14.52	-16.5
GO:0044597	GO Biological Processes	daunorubicin metabolic process	7	5.65	-15.23
GO:1901615	GO Biological Processes	organic hydroxy compound metabolic process	20	16.13	-14.26
GO:0018209	GO Biological Processes	peptidyl-serine modification	15	12.1	-14.1
GO:1904645	GO Biological Processes	response to amyloid-beta	10	8.06	-13.63
GO:0050878	GO Biological Processes	regulation of body fluid levels	17	13.71	-12.72
GO:0048511	GO Biological Processes	rhythmic process	15	12.1	-12.45
GO:1903829	GO Biological Processes	positive regulation of protein localization	18	14.52	-12.41
GO:0050727	GO Biological Processes	regulation of inflammatory response	17	13.71	-12.2
GO:0010942	GO Biological Processes	positive regulation of cell death	20	16.13	-12.18
GO:0043269	GO Biological Processes	regulation of ion transport	21	16.94	-12.14
GO:0080135	GO Biological Processes	regulation of cellular response to stress	21	16.94	-12.06
GO:0030335	GO Biological Processes	positive regulation of cell migration	19	15.32	-11.76
GO:0003013	GO Biological Processes	circulatory system process	18	14.52	-11.7

**Table 4:** List of tops enriched KEGG terms.

GO	Category	Description	Count	%	Log_10_(P)
hsa00910	KEGG Pathway	Nitrogen metabolism	12	9.68	-25.09
hsa05200	KEGG Pathway	Pathways in cancer	24	19.35	-17.64
hsa04151	KEGG Pathway	PI3K-Akt signaling pathway	17	13.71	-12.95
hsa04913	KEGG Pathway	Ovarian steroidogenesis	9	7.26	-12.19
hsa04914	KEGG Pathway	Progesterone-mediated oocyte maturation	10	8.06	-10.84
hsa04931	KEGG Pathway	Insulin resistance	9	7.26	-9.16
hsa00590	KEGG Pathway	Arachidonic acid metabolism	7	5.65	-8.22
hsa05206	KEGG Pathway	MicroRNAs in cancer	12	9.68	-8.2
hsa04072	KEGG Pathway	Phospholipase D signaling pathway	9	7.26	-7.95
hsa05010	KEGG Pathway	Alzheimer disease	11	8.87	-6.25
hsa04657	KEGG Pathway	IL-17 signaling pathway	6	4.84	-5.59
hsa04022	KEGG Pathway	cGMP-PKG signaling pathway	7	5.65	-5.22
hsa04360	KEGG Pathway	Axon guidance	7	5.65	-4.97
hsa05202	KEGG Pathway	Transcriptional misregulation in cancer	7	5.65	-4.82
hsa02010	KEGG Pathway	ABC transporters	4	3.23	-4.45
hsa04725	KEGG Pathway	Cholinergic synapse	5	4.03	-3.98
hsa00561	KEGG Pathway	Glycerolipid metabolism	4	3.23	-3.93
hsa04728	KEGG Pathway	Dopaminergic synapse	5	4.03	-3.66
hsa04971	KEGG Pathway	Gastric acid secretion	4	3.23	-3.56
hsa03410	KEGG Pathway	Base excision repair	3	2.42	-3.47

**Table 5:** List of tops enriched Hallmark terms.

GO	Category	Description	Count	%	Log_10(_P)
M5934	Hallmark Gene Sets	HALLMARK XENOBIOTIC METABOLISM	11	8.87	-9.15
M5901	Hallmark Gene Sets	HALLMARK G2M CHECKPOINT	8	6.45	-5.74
M5919	Hallmark Gene Sets	HALLMARK HEDGEHOG SIGNALING	4	3.23	-4.84
M5921	Hallmark Gene Sets	HALLMARK COMPLEMENT	7	5.65	-4.71
M5923	Hallmark Gene Sets	HALLMARK PI3K AKT MTOR SIGNALING	5	4.03	-4.13
M5915	Hallmark Gene Sets	HALLMARK APICAL JUNCTION	6	4.84	-3.74
M5937	Hallmark Gene Sets	HALLMARK GLYCOLYSIS	6	4.84	-3.74
M5950	Hallmark Gene Sets	HALLMARK ALLOGRAFT REJECTION	6	4.84	-3.74
M5946	Hallmark Gene Sets	HALLMARK COAGULATION	5	4.03	-3.57
M5935	Hallmark Gene Sets	HALLMARK FATTY ACID METABOLISM	5	4.03	-3.3
M5949	Hallmark Gene Sets	HALLMARK PEROXISOME	4	3.23	-3.04
M5947	Hallmark Gene Sets	HALLMARK IL2 STAT5 SIGNALING	5	4.03	-2.85
M5897	Hallmark Gene Sets	HALLMARK IL6 JAK STAT3 SIGNALING	3	2.42	-2.25
M5906	Hallmark Gene Sets	HALLMARK ESTROGEN RESPONSE EARLY	4	3.23	-2.03
M5945	Hallmark Gene Sets	HALLMARK HEME METABOLISM	4	3.23	-2.03
M5909	Hallmark Gene Sets	HALLMARK MYOGENESIS	4	3.23	-2.03

**Figure 2:**
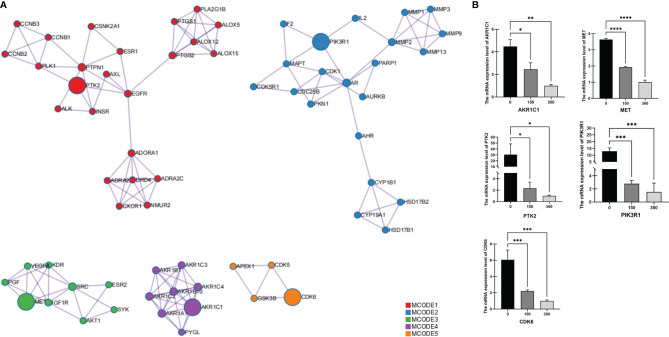
Identification of protein–protein interaction network hub genes for potential target proteins of *T. hemsleyanum*. **(A)** Hub gene screened by MCODE. **(B)** Quantitative PCR analysis confirmed that the aqueous extract of *T. hemsleyanum* was involved in the gene expression regulation of hub genes AKR1C1, MET, PTK2, PIK3R1, and CDK6. *p < 0.05, **p < 0.01, ***p < 0.001, ****p < 0.0001.

The MCODE algorithm in Cytoscape was then used to identify neighborhoods in which proteins were densely connected. Each MCODE network was assigned a unique color, as shown in **Figure 2A**. Five MCODEs were found in the PPI Network: MCODE1 (composed of 24 genes), MCODE2 (21 genes), MCODE3 (9 genes), MCODE4 (8 genes), and MCODE5 (4 genes). GO enrichment analysis was applied to each MCODE network to assign “meanings” to the network components; the three terms with the lowest p-values were retained and are shown in **Table 6**. Hub targets were the key nodes for evaluating the essence of the whole network and were identified by MCODE and MCC algorithms. AKR1C1, MET, PTK2, PIK3R1, and CDK6 were identified as the crucial hub targets in the network.

**Table 6:** Top three enrichment terms of each MCODE components in PPI network.

Network	GO	Annotation	Log_10_(P)
MCODE1	GO:0045859	regulation of protein kinase activity	-15.5
	GO:0051347	positive regulation of transferase activity	-14.8
	GO:0033674	positive regulation of kinase activity	-13.7
MCODE2	GO:0034644	cellular response to UV	-12.6
	GO:0071482	cellular response to light stimulus	-11.3
	GO:0009411	response to UV	-11.0
MCODE3	hsa04510	Focal adhesion	-13.6
	ko04510	Focal adhesion	-13.6
	WP306	Focal Adhesion	-13.5
MCODE4	GO:0044597	daunorubicin metabolic process	-25.0
	GO:0044598	doxorubicin metabolic process	-25.0
	GO:0030638	polyketide metabolic process	-25.0
MCODE5	GO:0021766	hippocampus development	-7.2
	GO:0021761	limbic system development	-6.8
	GO:0021543	pallium development	-6.2

To verify the accuracy of the network pharmacology and PPI analysis, we used experimental means to determine whether an aqueous extract of *T. hemsleyanum* could change the expression levels of the hub genes. As shown in **Figure 2B**, Q-PCR analysis demonstrated, for all concentrations of the aqueous extract of *T. hemsleyanum*, that the expression levels of each hub gene decreased significantly compared with the control group and that they decreased significantly along a gradient with increased concentration. These findings corroborate the *in silico* analysis and indicate that *T. hemsleyanum* significantly inhibits the expression of the target proteins.

### Gene Expression and Prognosis Analysis of Potential Target Proteins for *T. hemsleyanum* in Pan-Cancer

**Figure 3:**
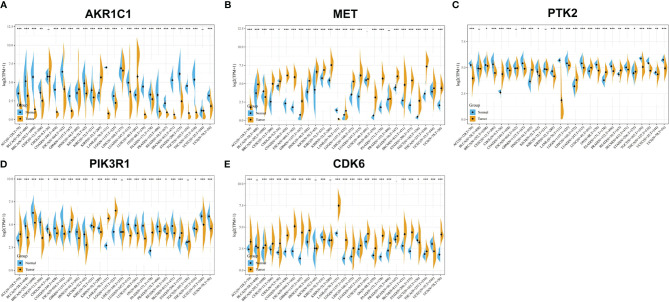
Gene expression analysis of potential target proteins of *T. hemsleyanum* in pan-cancer. **(A)** AKR1C1. **(B)** MET. **(C)** PTK2. **(D)** PIK3R1. **(E)** CDK6. *p < 0.05, **p < 0.01, ***p < 0.001.

**Figure 4:**
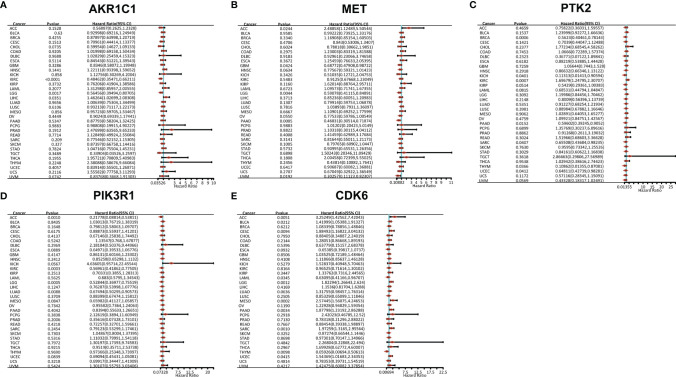
Gene prognostic analysis of potential target proteins of *T. hemsleyanum* in pan-cancer. **(A)** AKR1C1. **(B)** MET. **(C)** PTK2. **(D)** PIK3R1. **(E)** CDK6.

We used R software to calculate the difference in AKR1C1 expression between normal samples and tumor samples for each tumor and used non-paired Wilcoxon rank sum and signed rank tests to determine significance, as shown in **Figure 3A**. Significant up-regulation was observed in three tumors—HNSC, KIRP, and LUSC—and significant down-regulation was observed in 23 tumors—ACC, BLCA, BRCA, CESC, COAD, ESCA, GBM, KICH, KIRC, LAML, LGG, LIHC, LUAD, LUSC, OV, PAAD, PRAD, READ, SKCM, STAD, TGCT, THCA, and UCS. However, the expression of AKR1C1 in a univariate Cox prognostic analysis was inconsistent with its expression in tumors and normal tissues, as shown in **Figure 4A**. Generally speaking, proto-oncogenes are highly expressed in tumor tissues, which predicts poor prognosis and vice versa for tumor suppressor genes. In the PTK2 and PIK3R1 genes, the results were the reverse (**Figures 3C, D**, **4C, D**), so in the following analysis, these contradictory results are excluded.

**Figure 5:**
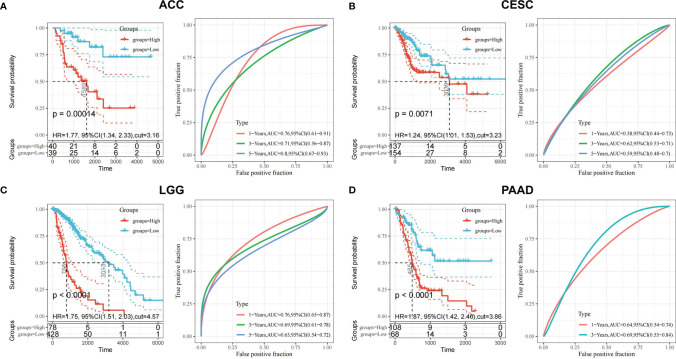
Prognostic validation of *T. hemsleyanum* potential target protein CDK6 in four tumors. **(A)** Kaplan–Meier survival analysis of CDK6 in ACC and ROC curve. **(B)** CESC. **(C)** LGG. **(D)** PAAD.

After screening all the data corresponding to expression and prognosis, it was determined that CDK6 and MET were potential target proteins for *T. hemsleyanum* and were significantly up-regulated in ACC (**Figure 3B**), whose high expression leads to poor prognosis (**Figure 4B**); CDK6 also showed similar results in CESC, LGG, and PAAD (**Figures 3E**, **4E**). We therefore chose CDK6 as the focus of downstream analysis. To verify the cancer-promoting effect of CDK6 in four tumors (ACC, CESC, LGG, and PAAD), Kaplan–Meier survival analysis was used to determine that CDK6 is not beneficial for patient prognosis and the ROC curve was used to characterize the relationship between the expression level of CDK6 and the prognosis—that is, the accuracy of the CDK6 model used to evaluate the survival of tumor patients. As shown in **Figure 5**, survival analysis for ACC showed that the high expression of CDK6 led to a poor prognosis for patients (HR = 1.77, p <.001), and the area under the curve (AUC) at 1, 3, and 5 years was greater than 0.7, indicating that the expression of CDK6 can be confidently used to predict adverse survival outcomes of ACC patients. Similarly, for CESC, Kaplan–Meier survival analysis also showed that the high expression of CDK6 led to poor prognosis for patients (HR = 1.24, p <.01), but the AUC at 1, 3, and 5 years was less than 0.7, indicating that the model of CDK6 expression used to predict clinical outcomes for patients in CESC has limitations. For LGG (HR = 1.75, p <.0001) and PAAD (HR = 1.87, p <.001), CDK6 led to a poor prognosis for patients, and the AUC was around 0.7, indicating that it has potential as a biomarker for the clinical prognosis of these tumors.

### Pan-Cancer Analysis of the Relationship Between the *T. hemsleyanum*-Targeted Protein CDK6 and Immunity

**Figure 6:**
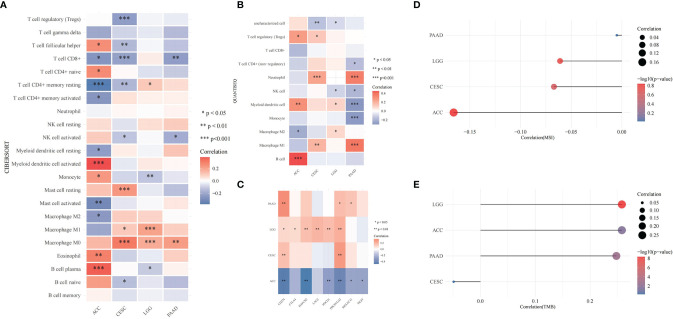
Correlation of *T. hemsleyanum*-targeted protein CDK6 with immunity and mutation of four tumors. **(A)** Analysis of tumor immune cell infiltration by CIBERSORT. **(B)** Analysis of tumor immune cell infiltration by quanTIseq. **(C)** Correlation analysis of immune checkpoints. **(D)** Correlation analysis of microsatellite instability. **(E)** Correlation analysis of TMB. *p < 0.05, **p < 0.01, ***p < 0.001.

**Figure 7:**
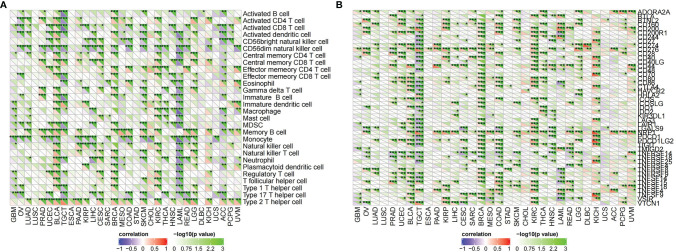
Correlation of *T. hemsleyanum*-targeted protein CDK6 with immunity in pan-cancer. **(A)** Analysis of tumor immune cell infiltration. **(B)** Correlation analysis of immune checkpoints. *p < 0.05, **p < 0.01, ***p < 0.001.

**Figure 8:**
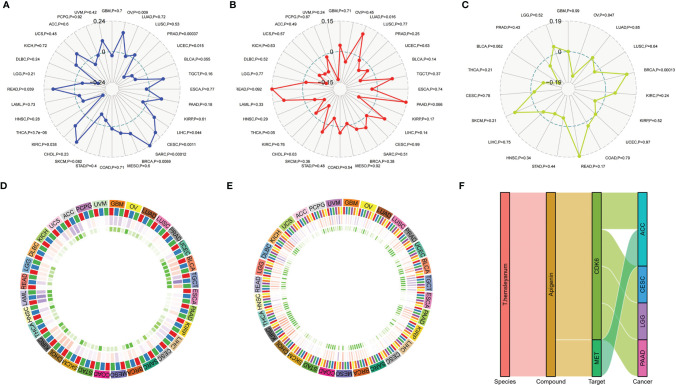
Correlation of *T. hemsleyanum*-targeted protein CDK6 with immunity and mutation in pan-cancer. **(A)** Correlation analysis of microsatellite instability. **(B)** Correlation analysis of TMB. **(C)** Correlation analysis of tumor neoantigens. **(D)** Correlation analysis of immune microenvironment. **(E)** Correlation analysis of tumor immune cell infiltration in pan-cancer. **(F)** Sanky plot of antitumor activity of *T. hemsleyanum*.

CDK6-related tumor immune infiltration analysis using the CIBERSORT and quanTIseq methods produced different results. To ensure the reliability of the analysis, this study only discusses tumors and immune cells for which similar results were obtained from both methods. For ACC, the infiltration of B cells (B cell plasm) and dendritic cells (myeloid dendritic cell resting) was positively correlated with the expression of CDK6, while the infiltration of M2 macrophages was negatively correlated (**Figures 6A, B**, **8E**). At the pan-cancer level, the effect of CDK6 on tumor immune cell infiltration in different tumors is very heterogeneous. For example, in LUSC, ESCA, STAD, CHOL, DLBC, and PCPG tumors, the expression of CDK6 is not related to the infiltration and distribution of most immune cells, but in BLCA, TGCT, BRCA, KIRC, LAML, and ACC tumors, the relationship is significant (**Figure 7A**).

An analysis of the correlations between the expression of CDK6 and immune checkpoints such as SIGLEC15, IDO1, CD274 (PD-L1), HAVCR2, PDCD1 (PD-1), CTLA4, LAG3, and PDCD1LG2 (PD-1) shows that the expression of CDK6 in ACC is negatively correlated with the transcripts of most of the immune checkpoint genes, while it is positively correlated with most such genes in LGG. For PAAD and CESC, the expression of CDK6 in ACC was not related to the transcript expression of most immune checkpoint genes (**Figure 6C**). A comprehensive evaluation of the correlations between CDK6 and tumor immune checkpoint genes at the pan-cancer level shows that CDK6 is similar in its infiltration of tumor immune cells. CDK6 is not related to the expression levels of immune checkpoint genes in LUSC, ESCA, STAD, and CHOL, but it is related to tumors such as BLCA, TGCT, BRCA, and KIRC, indicating that, from the perspective of immunity, the two analyses are synergistic (**Figure 7B**).

At the level of the immune microenvironment, the top three tumors are, according to the StromalScore scoring rules, BRCA (R = .369, p <.001), BLCA (R = .36, p <.001), and KIRC (R = .297, p <.001); according to ImmuneScore, they are BLCA (R = .36, p <.001), BRCA (R = .369, p <.001), and LAML (R = -0.326, p <.001); and according to ESTIMATEScore, they are BLCA (R = .36, p <.001), BRCA (R = .369, p <.001), and KIRC (R = .297, p <.001) (**Figure 8D**).

### Pan-Cancer Analysis of the Relationship Between *T. hemsleyanum*-Targeted Protein CDK6 and Mutation

In the microsatellite instability analysis, it was found that all four tumors for which CDK6 indicated a poor prognosis were negatively correlated with MSI; that is, CDK6 promotes the stability of the cell chromatin genome. Therefore, inhibiting the activity of CDK6 cells may affect the survival of tumor cells by inducing instability in the cell genome. The four tumors, in descending order of the magnitudes of their correlations with MSI are ACC, CESC, LGG, and PAAD (**Figure 6D**). At the pan-cancer level, the tumors with positive correlations between CDK6 expression and MSI are READ, KIRC, BRCA, SARC, CESC, LIHC, and OV and the tumors with negative correlations are THCA, UCEC, and PRAD (**Figure 8A**).

In terms of TMB, the expression of CDK6 was related to LGG, ACC, and PAAD, while CESC had little relationship with TMB (**Figure 6E**). At the pan-cancer level, LUAD was positively correlated with TMB, while THCA was negatively correlated. TMB is related to tumor neoantigen (**Figure 8B**); and generally speaking if TMB increases, patients can often benefit from targeted therapy, which is related to the generation of tumor neoantigen. At the pan-cancer level, in BRCA, the expression level of CDK6 was positively correlated with tumor neoantigen and negatively correlated with OV, showing that the roles and functions of CDK6 vary in different tumor mutations (**Figure 8C**). In general, we confirmed the potential pattern of anti-tumor activity of Tetrastigma hemsleyanum through a series of bioinformatics analysis, as shown in **Figure 8F**.

### Biological Function Identification of *T. hemsleyanum*-Targeted Protein CDK6 in Pan-Cancer

**Figure 9:**
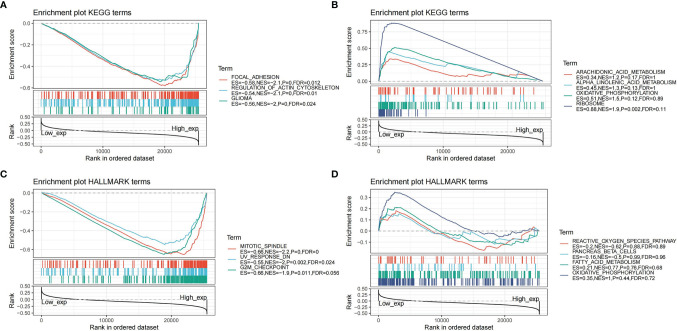
Gene set enrichment analysis in pan-cancer. **(A)** Enrichment plot of KEGG terms in high expression of CDK6. **(B)** Enrichment plot of KEGG terms in low expression of CDK6. **(C)** Enrichment plot of Hallmark terms in high expression of CDK6. **(D)** Enrichment plot of Hallmark terms in low expression of CDK6.

To study the function of CDK6 expression in pan-cancer, we divided the human pan-cancer samples into high and low expression groups according to the expression levels of CDK6 and we analyzed the enrichment of signal pathways in KEGG and Hallmark in both groups using GSEA. The three signal pathways most significantly enriched in the two databases in both groups are shown in **Figure 9A**. For the KEGG high expression group, focal adhesion (NES = -2.1, p <.001, FDR = .012), regulation of actin cytoskeleton (NES = -2.1, p <.001, FDR = .01), and glioma (NES = -2, p <.001, FDR = .024) were significantly enriched. For the KEGG low expression group, ribosome was the only significant enrichment pathway (NES = 1.9, p <.002, FDR = .11) (**Figure 9B**). For the Hallmark high expression group, mitotic spindle (NES = -2.2, p <.001, FDR <.001) and UV response (NES = -2, p = .002, FDR = .024) are related to the biological function of the G2M checkpoint (NES = -1.9, p = .011, FDR = .056) (**Figure 9C**), while for the Hallmark low expression group, no significant biological processes or signal pathways were enriched (**Figure 9D**).

## Discussion

CDK6 has been shown to play an important role in regulating cell cycle progression and the up-regulation of CDK6 activity is closely related to the occurrence and development of multiple types of cancer, including breast cancer, hematological malignancies, and several solid tumors. CDK6 is overexpressed in cancer cells but only detected at low levels in non-cancer cells and CDK6-null mice have been found to develop normally (11), suggesting a potential low-toxicity therapeutic strategy through the inhibition of the expression of CDK6. Selective small-molecule CDK6 inhibitors therefore have tremendous potential as antitumor drugs and we confirmed herein the inhibitory effects of *T. hemsleyanum* extract on the expression of CDK6.

In general, CDK6 participates in the protection of cell genome stability and prevents cell mutations, which could lead to tumorigenesis (20) and similar results were found in the present study: *T. hemsleyanum* specifically inhibits the expression levels of CDK6, making it easy to cause genomic damage and to kill tumor cells, especially adrenocortical carcinoma, cervical cancer, low grade gliomas, and pancreatic cancer. The TMB results underline the biological function of CDK6 in maintaining cell stability. In previous research, overexpression of DNA damage and cell cycle–dependent proteins were observed to be associated with poor survival in 79 adrenocortical carcinoma patients and were accompanied by the significant up-regulation of genes involved in DNA damage and the regulation of cell cycle pathways; indeed, greater expression of CDK6 was associated with worse survival irrespective of age or sex (21).

Notably, tumor suppressor p16INK4A is indispensable for the survival of cervical carcinoma cell lines and oncogenic p16INK4A activity depends on the inhibition level of CDK6 (22). Similarly, miR-145 overexpression has been found to inhibit the proliferative ability of human cervical carcinoma cells by downregulating the expression of CDK6 (23), and abnormally expressed microRNA is closely associated with pancreatic cancer, with miR-3613-5p having been found to increase the metastasis of pancreatic cancer by targeting CDK6 (24). Conversely, sequential treatment with CDK6 inhibitors following DNA-damaging chemotherapy was shown to improve the therapeutic effect in pancreatic cancer, which was due to the action of CDK6 inhibitors on the homologous recombinant proteins responsible for repairing chromosome damage (25).

Mesenchymal–epithelial transition tyrosine kinase receptor (MET or c-MET) is a high-affinity proto-oncogene receptor tyrosine kinase encoded by the MET proto-oncogene. Hepatocyte growth factor (HGF) is the native peptide ligand of the MET receptor and aberrant HGF/MET activation drives oncogenic pathways involved in the development and progression of several human cancers, including renal, gastrointestinal, lung, and breast carcinomas, as well as glioblastoma multiforme (GBM) (26, 27). MET mutation and/or amplification is often found in genitourinary malignancies and has been shown in phase I clinical trials to lead to worse prognoses (28). Recent studies have indicated that many tumors display MET/HGF pathway abnormalities, which can be seen from c-MET overexpression and from MET mutation and/or amplification. Drugs that target c-MET may offer a new strategy for the control of aggressive cancers and the combination of HGF/MET inhibitors with conventional chemotherapy in patients affected by different cancer types has shown promising results. In studies of liver cancer cell lines and mice with orthotopic tumors, MET inhibitors were shown to promote liver tumor evasion of the immune response by stabilizing PDL1 (29). The MET receptor has thus emerged as a druggable target across several human cancers and agents targeting MET and HGF, including small molecules such as crizotinib, tivantinib, and cabozantinib, have shown therapeutic effects in different tumors (30). In the present study, we demonstrated the inhibitory effects of *T. hemsleyanum* extract on the expression of MET. The active constituents of *T. hemsleyanum* are therefore promising candidates for improving the clinical activity of MET inhibitors and have the potential to improve survival rates and enhance therapeutic efficacy in human cancers.

Natural products generally have a variety of action targets and their biological processes and signal pathways are similarly multiple. In contrast, many widely used targeted drugs, such as immune checkpoint inhibitors or metabolic checkpoint inhibitors, may induce drug resistance in tumor cells as the target-sensitive main clone created by tumor heterogeneity gives way to non-sensitive subclones, gradually inducing drug resistance under the effect of mutation-screening caused by the targeted drugs. The multi-target effect of natural products therefore has the potential to overcome some of the drug resistance of targeted drugs and is likely to either trigger synergistic death in the process of drug use or to sensitize the targeted drugs in combination with the primary chemotherapy regimen, thus reducing drug toxicity and improving pharmacokinetics. Through the integrated network pharmacology pan-cancer analysis of the effective components of *T. hemsleyanum*, this study has shown that *T. hemsleyanum* acts as a cell cycle checkpoint inhibitor and a tyrosine kinase receptor inhibitor and has demonstrated the multi-target nature of *T. hemsleyanum* as a natural product, which was verified by bioinformatics analysis combined with experiments. The pivotal genes CDK6 and MET of the *T. hemsleyanum*-inhibited tumor were confirmed, providing a new theoretical basis for the clinical application of *T. hemsleyanum*.

In fact, many studies have already reported the antitumor effects of *T. hemsleyanum*, both *in vitro* and *in vivo*, and it has been widely used in clinics as an adjuvant drug to chemotherapy treatment. In this study we explored, for the first time, the antitumor mechanisms of *T. hemsleyanum* through network pharmacology combined with pan-cancer analysis. As a common screening method for natural product action targets, network pharmacological analysis relies on an existing database for text mining. Combined with pan-cancer analysis—as a means of comprehensively describing biological functions—and with phenotypic correlation analysis of molecules in different tumors, it represents a new strategy for the study of the antitumor activity of natural products.

## Data Availability Statement

The original contributions presented in the study are included in the article/**Supplementary Material**. Further inquiries can be directed to the corresponding author.

## Author Contributions

XP designed the project and experiment, CGW, YXZ, XDC, TJ and YS have completed the analysis, CGW, TJ and YS completed the experiment, XP, YXZ and CGW wrote the draft of the manuscript, and all the authors have read the manuscript and approved the publication of the manuscript.

## Funding

This research was supported by the Major Science and Technology Projects of Breeding New Varieties of Agriculture in Zhejiang Province (2021C02074) and by the Ningbo City Science and Technology Innovation 2025 Major Research Project (2019B10008).

## Conflict of Interest

CGW and YXZ were employed by Biotrans Technology Co., LTD.

The remaining authors declare that the research was conducted in the absence of any commercial or financial relationships that could be construed as a potential conflict of interest.

## Publisher’s Note

All claims expressed in this article are solely those of the authors and do not necessarily represent those of their affiliated organizations, or those of the publisher, the editors and the reviewers. Any product that may be evaluated in this article, or claim that may be made by its manufacturer, is not guaranteed or endorsed by the publisher.
